# Genome Sequence of Marine-Derived *Streptomyces* sp. Strain F001, a Producer of Akashin A and Diazaquinomycins

**DOI:** 10.1128/MRA.00165-19

**Published:** 2019-05-09

**Authors:** Jana Braesel, Chase M. Clark, Kevin J. Kunstman, Stefan J. Green, Mark Maienschein-Cline, Brian T. Murphy, Alessandra S. Eustáquio

**Affiliations:** a Department of Medicinal Chemistry and Pharmacognosy and Center for Biomolecular Sciences, College of Pharmacy, University of Illinois at Chicago, Chicago, Illinois, USA; b Sequencing Core, University of Illinois at Chicago, Chicago, Illinois, USA; c Research Informatics Core, University of Illinois at Chicago, Chicago, Illinois, USA; University of Delaware

## Abstract

We report the 9.7-Mb genome sequence of *Streptomyces* sp. strain F001, isolated from a marine sediment sample from Raja Ampat, Indonesia. F001 produces diazaquinomycins, which exhibit potent and selective antituberculosis activity. In addition, it is also known to produce akashin A, a blue pigment that has shown cytotoxic activity.

## Abstract (precis)

We report the 9.7-Mb genome sequence of *Streptomyces* sp. strain F001, isolated from a marine sediment sample from Raja Ampat, Indonesia.

## ANNOUNCEMENT

*Streptomyces* sp. strain F001 was isolated from a sediment sample from Raja Ampat, Bird’s Head, Papua, Indonesia. The marine-derived actinomycete strain F001 is known to produce two compound classes of biomedical interest, the chlorinated indigo glycoside akashin A (1) and several congeners of the diazaquinomycin class of antibiotics (2). Akashin A has demonstrated cytotoxic activity against cancer cell lines (3), whereas it was recently reported that some diazaquinomycin analogs exhibited potent and selective inhibitory activity against a panel of drug-resistant Mycobacterium tuberculosis strains (4).

Strain F001 was grown in International *Streptomyces* Project medium 2 (ISP2) liquid medium (0.4% yeast extract, 1% malt extract, 0.4% dextrose [pH 7.3]) for 3 days at 30°C and 200 rpm, and total genomic DNA was isolated using the Qiagen blood and cell culture DNA midi kit. The genome was sequenced using a combination of short-read (Illumina) and long-read (Pacific Biosciences) technologies. Genomic DNA was prepared for shotgun sequencing using a Nextera XT DNA library preparation kit (Illumina, San Diego, CA, USA), and the library was sequenced using an Illumina NextSeq 500 instrument employing paired-end 150-bp reads. Illumina sequencing yielded 4,790,310 reads. No quality control filtering of Illumina data was performed. The isolated high-molecular-weight DNA was also subjected to next-generation sequencing on the Pacific Biosciences (PacBio) single-molecule real-time (SMRT) DNA sequencing system as per the manufacturer’s protocols. Briefly, SMRTbell libraries were created with the Pacific Biosciences template preparation kit and sequencing protocol for 20-kb libraries. Sequencing was performed on the PacBio RS II system, and read filtering was performed with the PacBio SMRT Analysis software package using default settings. PacBio sequencing yielded 43,531 reads, and the read *N*_50_ value was 12,826 bp. *De novo* coassembly of Illumina and PacBio reads was performed using SPAdes 3.5.0 (5) with default parameters. Coverage levels were assessed by mapping raw Illumina reads back to the contigs with Bowtie 2 (6) and computing the coverage as the number of reads aligning per contig times the length of each read divided by the length of the contig. The average sequencing coverage was 47-fold.

The final assembly contains 171 contigs with a total size of 9,724,482 bp, an average G+C content of 70.3%, and an *N*_50_ contig length of 601,559 bp. Automatic functional annotation results were obtained using the Rapid Annotations using Subsystems Technology (RAST) Web server (7 – 9) under the following settings: annotation scheme, ClassicRAST; preserve gene calls, no; automatically fixed errors, yes; fix frameshifts, yes; backfill gaps, yes. A total of 8,981 protein-coding genes, 18 rRNA genes, and 73 tRNA genes were predicted. The genome sequence of *Streptomyces* sp. strain F001 will contribute to the identification of genes encoding akashin A and diazaquinomycin biosynthesis, in addition to potentially revealing additional biosynthetic gene clusters.

### Data availability.

The raw sequence reads have been submitted to the SRA under the number SRX5324109. The assembled genome sequence of *Streptomyces* sp. F001 has been deposited in GenBank under the accession number QZWF00000000. The version described in this paper is version QZWF01000000.
